# Editorial: Biosecurity of infectious diseases in veterinary medicine

**DOI:** 10.3389/fvets.2025.1665683

**Published:** 2025-08-01

**Authors:** Joel Fernando Soares Filipe, Maria Giovanna Ciliberti, George Attard, Gabriele Ratti, Mara Silvia Laura Rocchi

**Affiliations:** ^1^Department of Veterinary Medicine and Animal Sciences, University of Milan, Lodi, Italy; ^2^Department of Agriculture, Food, Natural Resources and Engineering Sciences, University of Foggia, Foggia, Italy; ^3^Department of Rural Sciences and Food Systems, University of Malta, Msida, Malta; ^4^I-Vet srl, Brescia, Italy; ^5^Virus Surveillance Unit, Moredun Research Institute, Penicuik, United Kingdom

**Keywords:** biosecurity, infectious diseases, One Health, antimicrobial resistance (AMR), risk assessment

The global animal health landscape is constantly under threat from new and re-emerging infectious diseases. Apart from the negative impacts on animal welfare and the decrease in livestock productivity, they also pose serious risks to public health through the transmission of diseases from animals to humans. Effective biosecurity is crucial in preventing the introduction and spread of pathogens within and between animal populations. This Research Topic explores various aspects of biosecurity in veterinary medicine, focusing on new approaches, critical evaluations, and the complex interplay among human behavior, policies, and technologies. The contributing articles collectively aim to improve the management of infectious disease risks, thereby protecting animal health, food security, and public health under the “One Health” approach.

At the core of any successful biosecurity program there are strict cleaning and disinfection (C&D) protocols. While evaluating these procedures is essential, it can also be challenging. One of the studies included in this topic reviewed different methods for assessing C&D, including visual inspections, ATP bioluminescence, microbiological and molecular analyses, emphasizing the need for a comprehensive, individualized approach to ensure effective hygiene management (Makovska et al.). Additionally, research into new disinfection agents offers promising alternatives. For example, a study evaluating chlorous acid water as a disinfectant used at the pre-surgically stage in cattle found it to be as effective and comparable to standard approved methods, potentially decreasing preparation time in field settings (Ichii et al.). Another research explored the efficacy of UV254 irradiation for inactivating major swine viruses like African Swine Fever Virus (ASFV), Porcine Reproductive and Respiratory Syndrome Virus (PRRSV), and Porcine Epidemic Diarrhea Virus (PEDV) in both water and air, providing useful information for decontamination of the environment on swine farms (Qiu et al.). These studies reflect the ongoing scientific work that has been done to improve and innovate the physical and chemical barriers against pathogen transmission.

Further emphasizing the element of human intervention, a survey of North American swine producers' biosecurity practices showed the dynamics affecting their adoption of biosecurity plans, varying according to farm size and level of perceived disease risk. This work highlights the need for updated assessments and the introduction of artificial intelligence systems, such as machine learning, for risk assessment evaluation, recognizing the role of demographics and risk perception in adoption (Chepkwony et al.).

Effective biosecurity involves more than just technical procedures, it is actually heavily influenced by human behavior, policy, and communication strategies, and because of these elements, it is essential that farmers are understood, and encouraged to engage. Another study from a research group of China provides valuable insights into how legislative regulation affects biosecurity investments by pig producers, showing that effective law enforcement and tailored regulations can significantly boost prevention efforts, especially among medium-scale farmers (Liu and Tao). In a different context, a pilot intervention in Tanzania demonstrated the effectiveness of a participatory approach in improving biosecurity practices on small and medium-scale pig farms. By collaboratively creating checklists and fostering cooperation between farmers and livestock field officers, significant improvements in overall farm production and biosecurity compliance were achieved, proving the effectiveness of grassroots, capacity-building strategies in areas with limited resources (Auplish et al.). Crucially, effective communication also emerges as a central theme. A study exploring stakeholder perspectives on communication methods for biosecurity advocates for collaborative, personalized, and sustainable approaches, emphasizing direct interaction, practical learning, and the integration of technological tools to promote behavioral change (Moya et al.). These articles as a whole illustrate that the effective implementation of biosecurity measures requires an integrated approach of the attitudes, knowledge, and cooperation of all stakeholders involved in the biosecurity assurance.

Disease-specific biosecurity intervention is essential for specific disease concerns. Infectious Bronchitis (IB) in poultry, for instance, is an infectious avian disease that can potentially lead to severe economic losses. An investigation of IB outbreaks on a broiler farm revealed that external and internal biosecurity deficits coupled with inappropriate vaccine selection facilitated the introduction and spread of wild viral strains, highlighting the extreme importance of good biosecurity practices (Maletić et al.). In dairy cattle, *Salmonella* Dublin poses an enzootic threat. A study from Denmark identified specific farm sections where lower biosecurity scores were associated with a higher risk of *S. Dublin* introduction and establishment, indicating that current biosecurity levels may be insufficient to counteract infection pressure from the surroundings (Pedersen et al.).

Furthermore, to address the limitations of existing biosecurity assessment tools, particularly for diverse farming structures, a new biosecurity risk assessment tool (BioscoreDairy) has been developed and optimized for pasture-based dairy farms in Ireland. This innovative tool combines a questionnaire on management practices with an audit of cattle movement records, providing enterprise-specific risk categorization and benchmarking capabilities (O Donovan et al.). Such tools are crucial to identify specific vulnerabilities and guiding targeted interventions.

Besides direct disease control, the threat of antimicrobial resistance (AMR) is an unresolved issue. A systematic review of Streptococcus infection in bovine mastitis in Ethiopia revealed an extremely alarming rate of *Streptococcus* spp., and high rates of resistance to widely prescribed antimicrobials like penicillin. This makes evidence-based risk management and strict antimicrobial use standards imperative to avoid AMR (Fenta et al.). Moreover, the characterization of canine circovirus, an emerging pathogen, emphasizes the necessity of understanding its genetic variability, risk of cross-species transmission, and diagnostic challenges starting from a One Health approach in consideration of its significance in animal and public health (Ferreira da Silva et al.).

This Research Topic captures the dynamic and multidimensional nature of biosecurity in veterinary medicine. From C&D efficacy and new disinfectants to the general impact of laws and farmer engagement, and from unique disease control strategies to general risk assessment tools and emerging pathogen surveillance, each article brings its contribution with important issues and knowledge.

The joint findings underscore the fact that effective biosecurity is not an unchanging concept but an evolving discipline that requires continuous novelty in technology, dynamic policy tools, different communication strategies, and cooperative comprehension of animal and human behavior. In conclusion, what emerges from this collection is that for the future, interventions in biosecurity will require a more concerted and multi-disciplinary approach against infectious disease and for the safeguarding of welfare and health of animals and humans across the globe within the context of One Health approach.

